# Mitochondrial genome sequence from *Anthocoris kerzhneri* (Hemiptera: Anthocoridae) and phylogenetic analysis

**DOI:** 10.1080/23802359.2018.1481781

**Published:** 2018-06-12

**Authors:** Xinxin Li, Nan Song, Haoguang Meng

**Affiliations:** College of Plant Protection, Henan Agricultural University, Zhengzhou, China

**Keywords:** Mitochondrial genome, *Anthocoris kerzhneri*, phylogenetic analysis

## Abstract

This study sequenced and analyzed the nearly complete mitochondrial genome of *Anthocoris kerzhneri*. This mitogenome is 16,018 bp long and consisted of 13 protein-coding genes, 20 tRNA genes and two rRNA genes. All protein-coding genes begin with the typical ATN codons and end with TAA or TAG codons. Phylogenetic analysis confirmed that the Reduviidae and Anthocoridae were polyphyletic groups. The Miridae and Tingidae had a sister group relationship.

The *Anthocoris kerzhneri* belongs to the family Anthocoridae, which is predaceous and recognized as an important natural enemy for biological control. The family Anthocoridae includes approximately 600 described species worldwide (Schuh and Stys 1991; Jung et al. 2010). To date, only two mitochondrial genomes have been sequenced from the family (Du et al. 2016; Hua et al. 2008). In this report, we determined the nearly complete mitochondrial genome of *A. kerzhneri*, which is the first from the genus *Anthocoris* of Anthocoridae.

Samples of *A. kerzhneri* were collected from city of Zhengzhou, China (the geospatial coordinates: 113.635°E, 34.723°N). A subset of samples has been deposited as vouchers with the Entomological Museum of Henan Agricultural University lot #EMHAU-2016-Zz071348. The genomic DNA was extracted by using the TIANamp Micro DNA Kit (TIANGEN BIOTECH CO., LTD, Beijing,China) following the manufacturer’s protocol. The extracted DNA was utilized for further library construction and sequenced by the Illumina HiSeq2500 platform (Illumina, San Diego, CA). The GenBank accession number of the newly sequenced mitogenome is MH223674.

The sequence of mitogenome of *A. kerzhneri* was nearly complete, with 16,018 bp length. The missing region was mainly located between *rrnS* and *trnQ*, which contained transfer RNA genes (*trnI*) and the partial control region. The mitogenome obtained includes the 13 protein-coding genes, 20 tRNA genes (i.e. *trnI* and *trnG* are missing) and two rRNA genes. The base composition of the mitochondrial gene was 43.3% A, 30.2% T, 16.1% C and 10.4% G, with significant A + T bias. The Leucine is the most frequently used amino acids, accounting for 14.47%. All protein-coding genes begin with the typical ATN codons (i.e. six with ATG, three with ATC, two with ATT and two with ATA). All protein-coding genes terminated with TAA codons except for *nad3*, *cob* and *nad1* having TAG codons.

The 20 tRNA genes have the typical cloverleaf secondary structure, except for *tRNA-Ser* (AGN) which lacks the dihydrouridine arm. The *rrnL* gene was determined between *trnL1* and *trnV*, with length of 1255 bp and A + T content of 76.2%. The *rrnS* gene was determined between *trnV* and control region, with a length of 807 bp and A + T content of 77.1%.

The maximum-likelihood (ML) analysis showed that the Reduviidae and Anthocoridae were both polyphyletic groups, with high node support values (Figure 1). In addition, our analysis confirmed the hypothesis that the Miridae and Tingidae had a sister-group relationship. This result was congruent with previous studies based on the morphological and molecular data (Tian et al. 2008; Schuh et al. 2009).

**Figure 1.**
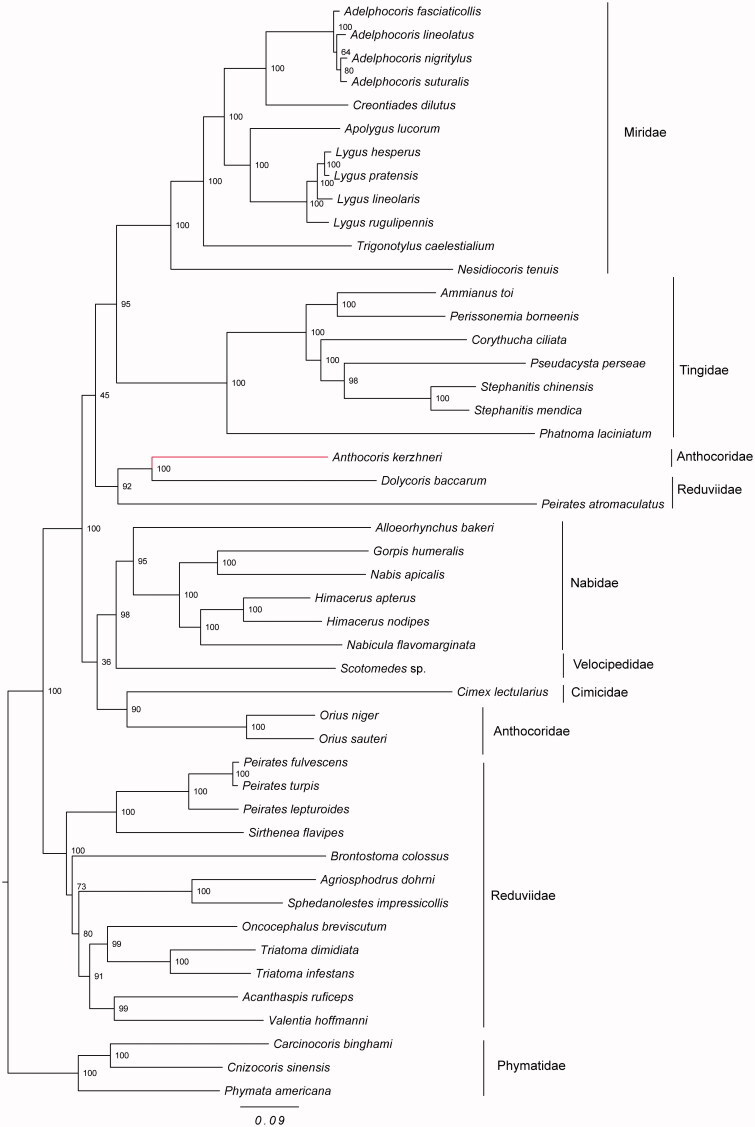
The maximum-likelihood tree was constructed using 13 protein coding genes from complete or near-complete mitochondrial genomes of A. kerzhneri and 46 other species. Phymatidae (Heteroptera: Cimicomorpha) was used to root the tree as an out-group. The accession numbers are as follows: Adelphocoris fasciaticollis (NC_023796); Adelphocoris lineolatus (KU234537); Adelphocoris nigritylus (NC_027144); Adelphocoris suturalis (KU234538); Creontiades dilutus (NC_030257); Apolygus lucorum (NC_023083); Lygus hesperus (NC_024641); Lygus pratensis (KU234540); Lygus lineolaris (NC_021975); Lygus rugulipennis (KJ170898); Trigonotylus caelestialium (KJ170899); Nesidiocoris tenuis (NC_022677); Ammianus toi (JQ739178); Perissonemia borneenis (KU896785); Corythucha ciliata (KC756280); Pseudacysta perseae (NC_025299); Stephanitis chinensis (MF498769); Stephanitis mendica (JQ739184); Phatnoma laciniatum (KU896786); Dolycoris baccarum (NC_020373); Peirates atromaculatus (NC_026670); Alloeorhynchus bakeri (HM235722); Gorpis humeralis (NC_019593); Nabis apicalis (NC_019595); Himacerus apterus (JF927831); Himacerus nodipes (JF927832); Nabicula flavomarginata (KX505851); Scotomedes sp. (JQ743677); Cimex lectularius (JQ739180); O. niger (EU427341); O. sauteri (NC_024583); Peirates fulvescens (NC_026669); Peirates turpis (NC_026671); Peirates lepturoides (NC_026672); Sirthenea flavipes (NC_020143); Brontostoma colossus (NC_024745); Agriosphodrus dohrni (NC_015842); Sphedanolestes impressicollis (KC887536); Oncocephalus breviscutum (NC_022816); Triatoma dimidiata(NC_002609); Triatoma infestans (NC_035547); Acanthaspis ruficeps (KX505848); Valentia hoffmanni (FJ456952); Carcinocoris binghami (NC_036012); Cnizocoris sinensis (NC_036013); Phymata americana (NC_036011).
